# Effect of supplementation with lutein, zeaxanthin, and omega-3 fatty acids on macular pigment and visual function in young adults with long-term use of digital devices: study protocol for a randomized double-blind placebo-controlled study

**DOI:** 10.3389/fnut.2024.1422468

**Published:** 2024-10-18

**Authors:** Lina Wang, Mei Ma, Yong Li, Cheng Pei, Jianming Wang, Juan Li, Linjuan Yang, Qianying Liu, Li Tang, Yang Hao, Huili Jiang, Jiaxuan Fu, Yuyao Xiao, Yahui Wang, Meng Cui, Tong Su, Jiaqi Bai, Hao Tang, Yue Wang, Hongying Shan, Hong Jiang, Chaoming Deng, Liyun Kong, Zhaozhao Hui, Le Ma

**Affiliations:** ^1^School of Public Health, Xi’an Jiaotong University Health Science Center, Xi’an, China; ^2^Shaanxi Eye Hospital, Xi’an People’s Hospital (Xi’an Fourth Hospital), Affiliated Guangren Hospital, Xi’an Jiaotong University Health Science Center, Xi’an, China; ^3^The First Affiliated Hospital, Xi’an Jiaotong University Health Science Center, Xi’an, China; ^4^The Second Affiliated Hospital, Xi’an Jiaotong University Health Science Center, Xi’an, China; ^5^BYHEALTH Institute of Nutrition & Health, Guangzhou, China; ^6^Key Laboratory for Disease Prevention and Control and Health Promotion of Shaanxi Province, Xi’an, China; ^7^Key Laboratory of Environment and Genes Related to Diseases, Xi’an Jiaotong University, Ministry of Education of China, Xi’an, China

**Keywords:** vision impairment, young adults, lutein, zeaxanthin, omega-3 fatty acids, macular pigment, visual function, randomized controlled trials

## Abstract

**Background:**

Growing evidence emphasizes the importance of xanthophyll carotenoids and omega-3 fatty acids in eye health. However, the beneficial effects of such supplementation have not been thoroughly discussed among adults with high screen exposure. Current trial evidence on lutein bioavailability is contradictory, and the interactions of dietary intervention with host-related factors remain elusive. This study aims to investigate the comparative effectiveness of supplementation with macular xanthophylls and omega-3 fatty acids on macular pigment optical density (MPOD) and visual function, access the bioavailability of free lutein and lutein ester, and explore the complex interplay between genetic variations, intestinal microbiota, and the dietary intervention in Chinese adults with long-term exposure to digital devices.

**Methods:**

The Lutein, Zeaxanthin, and Omega-3 (LZO) clinical trial is a 24-week multicenter, randomized, double-blind, placebo-controlled trial of 600 participants recruited from research centers, universities, and communities. Individuals are eligible to participate if they are aged over 18 years and use digital devices for over 8 h daily in the last 2 years, and will be randomized to six arms. A total of three visits will be scheduled at baseline, 12 and 24 weeks. The primary outcome is the change in MPOD over the 24-week intervention. The secondary outcomes are changes in visual function (visual acuity, best-corrected visual acuity, contrast and glare sensitivity, critical flicker fusion, reaction time, visuognosis persistence, symptoms and signs of dry eye, retinal thickness, and optical quality), and changes in serum lutein and zeaxanthin concentrations, and erythrocyte membrane omega-3 fatty acids. Genetic variations will be determined using genome-wide genotyping at baseline. 16S rRNA gene sequencing will be utilized to assess microbiome compositional changes before and after intervention.

**Discussion:**

The trial is anticipated to establish early interventions to prevent photochemical ocular damage and delay the onset of vision impairment in young adults with long-term repeated exposure to screen-based electronic devices, and provide valuable insights for the development of precision nutrition strategies for maintaining eye health.

**Clinical trial registration:**

www.clinicaltrials.in.th, Identifier, TCTR20220904002.

## 1 Introduction

In 2020, at least 1.1 billion people worldwide were affected by vision impairment (VI) and this number is projected to reach 1.8 billion by 2050 (1–3). Despite the fact that 90% of cases associated with VI are preventable, the global annual economic cost of productivity losses was approximately $411 billion, which has attracted widespread attention (4). With rapid urbanization and the ubiquitous adoption of digital devices (e.g., computers, smartphones), the widespread application of screen-based activities has raised particular concerns about the undesirable impact of excessive digital display use on ocular health (5–7). Overexposure to blue light predisposes the photoreceptors and retinal pigment epithelium (RPE) cells to cumulative photochemical damage by oxidative stress, inflammatory responses, and mitochondrial apoptosis (8, 9). Although the blue light emitted from electronic devices is well below safe viewing limits and it is not intense enough to induce acute damage in the retina (10), the long-term exposure to the low-illuminance artificial light may have chronic, cumulative effects on eye health. Therefore, an early intervention to light-induced damage is needed to prevent the formation of incurable retinopathy and thereby reduce the risk of vision loss.

As the primary pigment region in the posterior pole of the retina, the macula lutea is characterized by preferential deposition of lutein and zeaxanthin [collectively termed macular pigment (MP)], and responsible for the sharpest and optimal spatial vision (11). These two xanthophyll carotenoids have been indicated to serve as blue light filters and antioxidants to protect the vulnerable photoreceptor cells against light-induced oxidative stress through protective mechanisms including the absorption of short-wavelength blue light, scavenging of free radicals and reactive oxygen species (ROS), and neutralization of photosensitizers, suggesting that they might have the capacity to prevent retinal lesions (12–15). Previous randomized clinical trials (RCTs) have demonstrated the benefits of macular xanthophylls for preventing the development and progression of age-related macular degeneration (AMD) (16–18). Existing data regarding the beneficial effect of supplementation with xanthophyll carotenoids on visual performance are largely restricted to healthy population and patients with AMD (19–21). However, their efficacy in people with long-term cumulative exposure to digital screens has not been thoroughly discussed (7, 22, 23). Given that the zeaxanthin: lutein ratio reaches a maximum in the fovea of the macula, the differential distributions of the two carotenoids imply that zeaxanthin may exert the specific effect on macular health and visual performance (24). Besides, the current knowledge on supplemental intake of free lutein and lutein esters in serum lutein concentrations and macular pigment optical density (MPOD) has shown inconsistent results (25–29). As the major structural lipid avidly retained in retinal photoreceptor outer segments (POS) (30, 31), omega-3 fatty acids [docosahexaenoic acid (DHA, C22:6n-3) and eicosapentaenoic acid (EPA, C20:5n-3)] have been postulated to confer protective roles in the normal growth and development of neurons and RPEs through pleiotropic mechanisms such as suppressing oxidative stress, modulating inflammation, and inhibiting angiogenesis (32, 33), all of which have been implicated in the etiology of AMD (34, 35). Nevertheless, it remains unknown whether supplemental intake of lutein, zeaxanthin and omega-3 fatty acids has synergistic effects on macular morphology and visual function (36, 37). Thus, such an RCT related to the additional consumption of omega-3 fatty acid supplements should be conducted to provide valuable insights on these associations. Furthermore, emerging evidence has shown that the wide-ranging interindividual variability in response to nutrient supplementation is particularly affected by multiple factors, including race, genetic variations, and gut health (38, 39); to date, the majority of previous randomized trials using similar interventions were conducted in Caucasian. The role of the genetic variation at specific genes and composition of the gut microbiome in modulating the effects of lutein, zeaxanthin, and omega-3 fatty acid supplements on the vision system are yet to be explored, especially in Asian populations.

To address these knowledge gaps, the multicenter clinical trial will be performed to evaluate the comparative effectiveness or potential additive effects of supplementation with lutein, lutein plus zeaxanthin, and the combination of lutein, zeaxanthin, and omega-3 fatty acids on MP and various visual functions in Chinese young adults with long-term use of digital devices. We also assess the bioavailability of lutein from free or ester forms, and determine the relationships between genetic variations, intestinal microbiota, and the dietary effects on macular health and visual performance. The trial will expand the existing knowledge about protective effects against ocular damage and precision nutrition interventions in those who are more sensitive to photochemical risk or more exposed to blue light emitted from digital devices.

## 2 Materials and methods

### 2.1 Study design and population

The Lutein, Zeaxanthin, and Omega-3 (LZO) trial is a 24-week multicenter, randomized, double-blind, double-dummy, placebo-controlled trial with six parallel arms in Xi’an, China. Participants will be recruited through flyers, posters, or newsletter listings in research centers, universities, and communities. The study protocol was approved by the institutional review board at the Xi’an Jiaotong University (2019–1288) and was registered on the Thai Clinical Trials Registry (TCTR20220904002). A full explanation about the purpose and protocol of the trial will be provided to each individual and written informed consent will be collected from all participants following the guidance from investigators prior to study enrollment.

### 2.2 Eligibility criteria

Participants are eligible for inclusion if they are aged 18 years or older and use digital devices for more than 8 h daily in the last 2 years, ascertained by self-reported data (detailed below). Individuals are excluded if they have the presence of ocular diseases (e.g., congenital retinal diseases, cataracts, glaucoma, macular edema, macular holes, central serous chorioretinopathy, diabetic retinopathy, amblyopia, and strabismus); have a prior history of intraocular surgeries (e.g., an implanted intraocular lens, laser treatment, photodynamic therapy, and retina-vitreous surgery); currently use with orthokeratology lenses for ametropy management; have a history of systemic diseases (e.g., diabetes mellitus, cardiovascular disease, cancer, chronic kidney disease, gastrointestinal disorders, hematologic, and metabolic diseases); are currently pregnant, lactating or scheduled for pregnancy in the next 6 months; are vegetarians; are taking any supplements containing ingredients of the test products within the 6 months before enrollment; concomitant participation in other studies involving dietary supplementation or blood sampling; and are unavailable to attend the scheduled follow-up appointments.

### 2.3 Randomization and masking

An independent statistician conducts block randomization via a computer-generated randomization sequence with permuted block sizes of 12 individuals. After completion of baseline assessments, eligible participants are randomized in a 1:1:1:1:1:1 ratio to receive either five nutritional supplements or matching placebos for 24 weeks. Test products and placebos are manufactured by BYHEALTH Co., LTD, and BASF Co., LTD. Each active supplement and corresponding placebo are identical to one another in appearance and odor. They are prepackaged identically in sealed opaque bottles and precoded with consecutive numbers confidentially. Study staff will dispense supplements to participants immediately according to the order in which they complete their baseline visit. Participants and investigators (e.g., researchers dispensing study supplements, research coordinator, and outcome assessors) remain blinded to the group allocation throughout the trial, with data analysts masked until the database is locked. Unblinding will be done when the analysis of the endpoints is completed.

### 2.4 Sample size calculation

The sample size calculation was determined by assuming a > 20% increase in MPOD change between the intervention arms receiving lutein and zeaxanthin supplementation and the placebo (19), if a 2-sided type I error and statistical power were set as 5 and 80%, respectively. A total of 75 participants should be recruited in each arm. Accounting for 20% potential dropouts, a minimum of 94 participants per group would be needed. Finally, the number of enrolled participants was determined to increase to 100 in each group (600 for 6 groups) for an adequate sample size.

### 2.5 Interventions

Eligible participants will be randomized to take one of the following supplements orally twice daily: (1) tablet 1 (3 mg of lutein ester and 1 mg of *β*-carotene) and placebo 1 (soybean oil); (2) tablet 2 (6 mg of lutein) and placebo 1; (3) tablet 3 (6 mg of lutein and 1.2 mg of zeaxanthin) and placebo 1; (4) tablet 4 (12 mg of lutein ester and 1.2 mg of zeaxanthin) and placebo 1; (5) tablet 3 and capsule 1 (130 mg of DHA plus 325 mg of EPA); and (6) placebo 1 and placebo 2 (microcrystalline cellulose). Capsule 1 and placebo 1 are provided as a clear slightly yellowish oil encapsulated in soft gel capsules, and placebo 2 is manufactured as tablets and is matched in appearance to those containing carotenoids (tablets 1–4). The composition of supplements is presented in Table 1. All the test products and matching placebos have undergone extensive quality control testing provided by the manufacturer, under quality assurance considering stability of nutrient content and other parameters, and the presence of microorganisms, heavy metals, and pesticide residue. Furthermore, the contents of active agents are verified for each batch. Participants will be instructed to take their supplements with or immediately after a meal, and record their daily intake of trial supplements in precoded recording sheets. They will be asked to maintain their routine diet and not to take additional carotenoids and omega-3 fatty acids from supplemental sources during the entire period of 24 weeks. The lowest dose corresponded to an approximate daily intake of lutein (0.06–4.84 mg/d) and *β*-carotene (0.1–8.8 mg/d) and could be used as a reference group (40). The dosage of 12 mg lutein daily was chosen based on our previous trials (22) and the extensive intervention studies (18, 41, 42). Lutein has been determined to be “Generally Recognized as Safe” by Food and Drug Administration, and the dose selection was well below the acceptable daily intake (60 mg/d) defined by the European Food Safety Authority (43). The ratio of lutein to zeaxanthin was designed on the basis of the Age-Related Eye Disease Study 2 trial (44). Group 4 was designed to replace 12 mg/d of free lutein with 24 mg/d of lutein ester to assess the bioavailability of lutein from free or ester forms. Given that the optimal ratio of EPA to DHA was unknown (45), we selected a 2.5:1 ratio of EPA to DHA and a total dose of marine omega-3 fatty acids of 0.91 g/d, which was lower than 1 g/d used in the Memory Investigation with Nutrition for Dementia trial (EPA to DHA ratio, 3.3:1) (46), the Age-Related Eye Disease Study 2 trial (EPA to DHA ratio, 1.86:1) (44), and Vitamin D3-Omega-3-Home exercise-Healthy aging and longevity trial (EPA to DHA ratio, 1:2) (47). The chosen dosage of omega-3 fatty acids was below 3 g/day set by Food and Drug Administration, and was recognized as safe (48).

**Table 1:** Composition of supplements tested in the current study (per tablet or capsule).

Group	Tablet	Capsule
1	3 mg of lutein ester and 1 mg of *β*-carotene	Placebo 1 (soybean oil)
2	6 mg of lutein	Placebo 1 (soybean oil)
3	6 mg of lutein and 1.2 mg of zeaxanthin	Placebo 1 (soybean oil)
4	12 mg of lutein ester and 1.2 mg of zeaxanthin	Placebo 1 (soybean oil)
5	6 mg of lutein and 1.2 mg of zeaxanthin	130 mg of DHA and 325 mg of EPA
6	Placebo 2 (microcrystalline cellulose)	Placebo 1 (soybean oil)

DHA, docosahexaenoic acid; EPA, eicosapentaenoic acid.

2 mg of lutein ester is equivalent to 1 mg of lutein.

### 2.6 Plans for participant retention and adherence

The comprehensive physical and ophthalmic examination will be provided free of charge for each participant to increase their motivation to participate in the research. Besides, individuals will be encouraged to contact investigators if they have any potential concerns regarding the contents of the LZO trial.

Adherence will be monitored by trained staff based on pill counts confirmed at the end of the treatment period. Supply of tablets and capsules will be distributed at 12-weekly intervals, and all bottles containing any unused tablets and capsules are required to return at the next visit. Compliance to assigned treatments is calculated as the number of pills consumed divided by the expected number provided, expressed as a percentage. Participants will be considered compliant if they take ≥80% of the prescribed supplements during the 24 weeks of follow-up. The integration of monetary incentives with regular phone calls and text message reminders will be done in an effort to encourage all randomized participants to continue take assigned supplements and reinforce their willingness to complete the study process. Each participant will be offered funds for their transportation and receive 100 CNY to thank them for the completion of this trial.

### 2.7 Follow-up procedures

**Figure 1:**
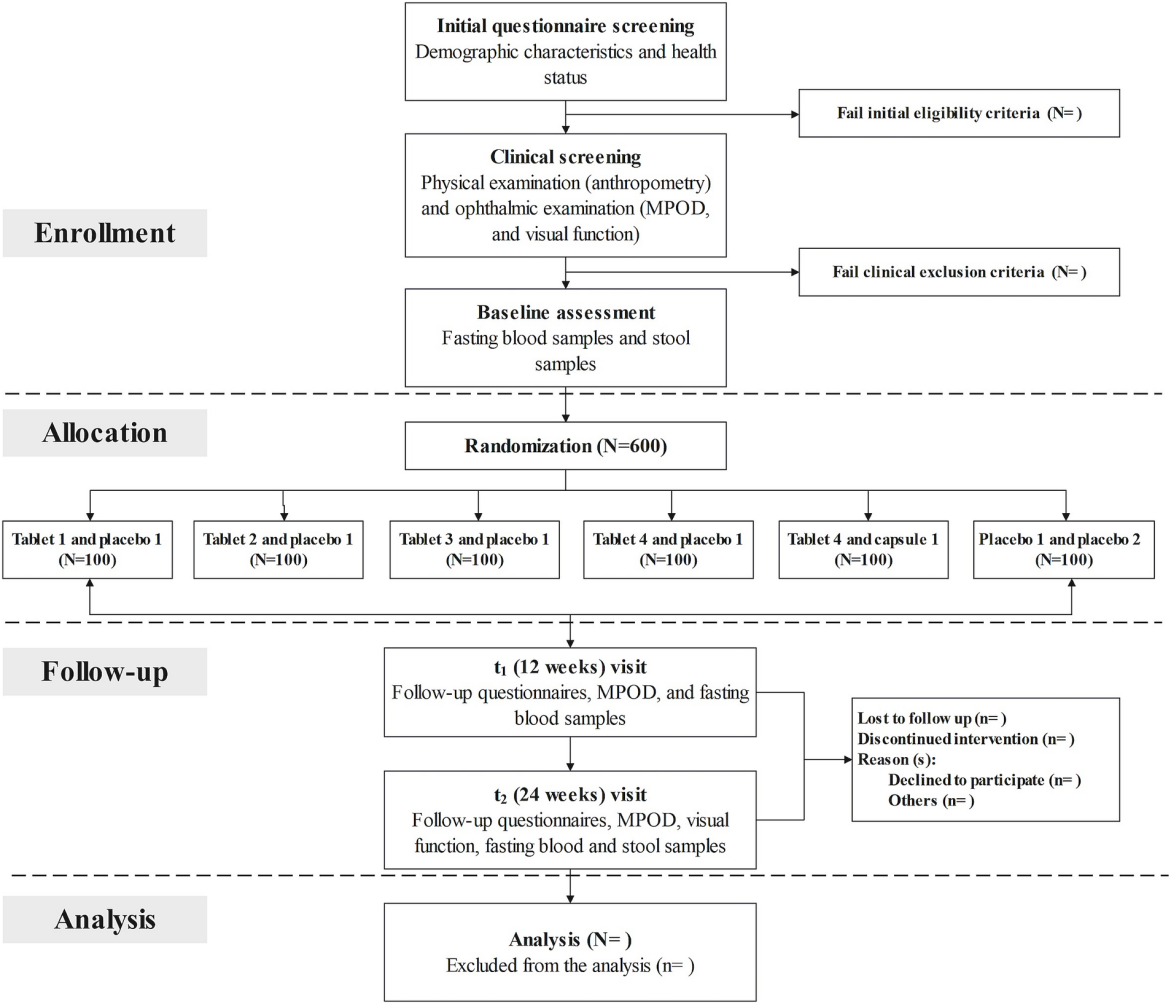
Flow chart of the clinical trial. MPOD, macular pigment optical density.

A total of three follow-up visits will be scheduled at baseline (prior to randomization), 12 weeks (secondary time point), and 24 weeks (primary time point). At enrollment, potential participants will be invited to the research center to provide informed consent and complete a screening questionnaire on demographic characteristics and health status, including information on lifestyle behaviors, preceding medical diagnoses as well as medications and dietary assessment. After successful completion of initial questionnaire screening, a routine clinical examination, including a physical examination and a comprehensive ophthalmic examination, will be performed in the hospital to further confirm their eligibility. Anthropometric measurements (e.g., height, weight, and waist circumference), evaluation of MPOD and visual function, and collection of biological samples (fasting blood and feces) will be fitted to each individual at the same time. At the 12-week visit, participants are asked to complete the follow-up questionnaire to provide update information on health status, and undertake assessments of MPOD and visual function. At the end of the 24-week supplementation period, they will be invited to return the research center, and scheduled to visit the hospital for repeated measurements. Fasting blood samples will be collected, and concentrations of serum lutein and zeaxanthin, and red blood cell (RBC) membrane omega-3 fatty acids will be measured at each study visit. Genome-wide genotyping will be assessed at baseline. Gut microbiota profiles will be performed for all fecal samples collected at baseline and the 24-week follow-up visit. The study staff will make regular telephone calls to participants to confirm their health condition and medication adherence. The time schedule of enrollment, interventions, assessments, and follow-up for participants is described in Figure 1 (flow chart of the clinical trial) and Table 2 (enrollment, interventions, and assessments).

**Table 2:** Schedule for enrollment, interventions, and assessments.

Time point	Study period			
	Enrollment	Allocation	Post-allocation	
	-t_1_	t_1_	t_2_	t_3_
		(Week 0)	(Week 12)	(Week 24)
Enrollment				
Eligibility screen	×			
Informed consent	×			
Allocation		×		
Interventions				
Tablet 1 and placebo 1		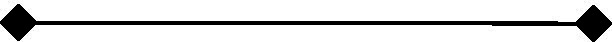		
Tablet 2 and placebo 1		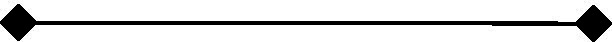		
Tablet 3 and placebo 1		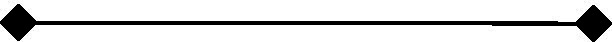		
Tablet 4 and placebo 1		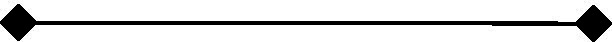		
Tablet 4 and capsule 1		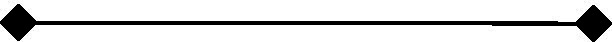		
Placebo 1 and placebo 2		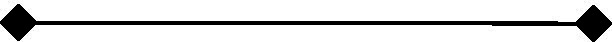		
Assessments				
Demographics	×			
Lifestyle behaviors	×		×	×
Medical history and medications	×		×	×
Dietary assessment	×		×	×
Anthropometry	×		×	×
MPOD	×		×	×
UCVA and BCVA	×		×	×
CS and GS	×		×	×
CFF	×		×	×
Reaction time	×		×	×
Visuognosis persistence	×		×	×
Symptoms and signs of DED	×			×
Retinal thickness	×			×
Optical quality	×			×
DNA extraction and genotyping		×		
Fasting blood (e.g., serum lutein and zeaxanthin, RBC membrane omega-3 fatty acids)		×	×	×
Stool samples for microbiome analysis		×		×
Provided supply of supplements		×	×	
Adverse events		×	×	×
Collect unused test product			×	×

t_1_, baseline; t_2_, at 12 weeks; t_3_, at 24 weeks; MPOD, macular pigment optical density; UCVA, uncorrected visual acuity; BCVA, best-corrected visual acuity; CS, contrast sensitivity; GS, glare sensitivity; CFF, critical flicker fusion; DED, dry eye disease; RBC, red blood cell.

### 2.8 Primary and secondary outcomes

The primary outcome is the change in MPOD over the course of the trial. Secondary outcomes include the change in the comprehensive test battery for visual function [best-corrected visual acuity (BCVA), CS, GS, critical flicker fusion (CFF), reaction time, visuognosis persistence, in-depth assessment of dry eye disease (DED) diagnosis and symptomatology, retinal thickness, and optical quality], changes in the concentrations of serum lutein and zeaxanthin, and erythrocyte membrane omega-3 fatty acids at each time point, the compositional changes in the intestinal microbiota before and after supplemental intake of lutein, zeaxanthin, and omega-3 fatty acids, and determination of whether changes in primary and secondary outcomes are associated with genetic variations, baseline microbiome characteristics, and changes in intestinal microbiota.

### 2.9 Data collection

#### 2.9.1 Macular pigment optical density

MPOD will be assessed at 0.5° retinal eccentricity for both eyes using the MPS 9000 (Macular Pigment Screener; Elektron Technology Corporation, UK). The method is determined psychophysically based on modified heterochromatic flicker photometry with excellent reliability and reproducibility as described in detail elsewhere (49, 50). Briefly, the amount of pigment at a distance of 0.5° from the fovea will be determined by a foveal target of 1° diameter located at 0.5° horizontal eccentricity with the reference spot located at 8° retinal eccentricity. A light stimulus of alternating blue light wavelength (460 nm) and green light wavelength (550 nm) at an individually-customized rate are superimposed on a uniformly illuminated white pedestal. Subjects are instructed to wear refractive correction spectacles, fixate on the central target, and respond to the flicker sensation by pushing a button as quickly as possible, but do not sacrifice accuracy for speed. The procedure of detecting flicker for a series of blue-green ratios in the fovea compared with that in the parafovea will be repeated and automatically recorded on the dedicated matching software of the equipment until a response curve is formed. The MPS II software subsequently calculates the MPOD value based on the curve, and automatically determines the value to three types of reliability (accept, caution, or reject); only if the plotted graph following a V shape with a clearly minimum will be considered as a successful test and judged as “accept” by the software. MPOD values will be expressed in optical density units and provided on a scale of 0–1. A successful practice run will be performed to familiarize all subjects with the flashing target location to improve the accuracy of the test prior to the formal test. Considering the interindividual differences in flicker sensitivity, a pretest flicker sensitivity routine (30 s) will be used to adjust the initial luminance contrast and the appropriate flicker sensitivity range during the test session. Measurement will be performed in triplicate by trained and certified study staff using standardized procedures, and the mean value of MPOD for each eye will be obtained.

#### 2.9.2 Visual function

##### 2.9.2.1 Visual acuity

VA without correction (UCVA) and best spectacle-corrected VA after diopter correction will be measured on each eye in duplicate using the Early Treatment of Diabetic Retinopathy Study (ETDRS) chart and standard operating procedures. More details on the device are described elsewhere (51). In brief, the participants are required to accurately recognize letters as more as possible at a distance of 4 meters in adequate room lights until 5 incorrect answers in a row are made. Acuity for each eye is recorded as the total number of letters identified correctly and then calculated as the logarithm of the minimum angle of resolution.

##### 2.9.2.2 Contrast and glare sensitivity

Contrast and glare sensitivity will be measured separately for each eye using the CSV-1000E (Vector Vision Corporation, USA) at photopic and mesopic illumination conditions, respectively. The standard operating procedures of the measurement are available in previous publications (52). Subjects are shown a series of achromatic sine-wave targets of four spatial frequencies (3, 6, 12, and 18 cycles per degree) presented on the rear illuminated chart placed 3 meters away. Each row consists of vertical target pairs of eight different contrast levels arranged in order. Following verbal instruction and narration, participants will be required to accurately identify the visual target of every spatial frequency as more as possible. The task terminates when correctly discrimination at different levels of spatial frequency is failed, and log contrast sensitivities are recorded by a trained investigator.

##### 2.9.2.3 Critical flicker-fusion frequency

CFF testing will be performed on both eyes using a CFF monitor, following the manufacturer’s instructions (53, 54). The test involves a discontinuous light stimulus fixed in the fovea binocular of the apparatus, and then the frequency of the target gradually increases until participants perceptually perceive the fusion (ascending frequency) or gradually decreases until they identify the visual flicker (descending frequency). Both ascending and descending trials are taken in duplicate to determine the mean threshold frequency.

##### 2.9.2.4 Reaction time

Reaction time testing is a quantitative method for assessing the speed and precision of visual processing systems (55). It is conducted with simple reaction time (SRT) and choice reaction time (CRT), following a standardized procedure by trained experienced investigators. The SRT task involves a participant who is instructed to press a button as quickly as possible when a visual stimulus is displayed at a random location on the screen of a specific device. The CRT test involves the visual stimuli of simple graphics presented on the linear light array. The participants are asked to respond correctly to match the stimulus as quickly as possible. The mean RTs of 40 test trials are recorded in milliseconds for each participant.

##### 2.9.2.5 Visuognosis persistence

A visuognosis persistence test is widely used to evaluate eye fatigue published by the China Food and Drug Administration (23). In brief, the subjects are instructed to stare at the cubes in a 3-dimensional block diagram at a distance of 1.5 m for 3 min. Unclear vision is defined as inverted cubes. Visuognosis persistence is calculated as the clear vision time divided by the total duration of gaze.

##### 2.9.2.6 Dry eye symptoms and signs

Severity of DED symptoms will be assessed using the Ocular Surface Disease Index (56), which consists of 12 items about DED in 3 categories: eye symptoms, visual-related function, and environmental stimulus factors, and ranges from 0 to 100, with higher scores denoting higher severity. This scale has been widely used in the general population and has been shown to be a valid and reliable instrument, appropriate for evaluating the subjective impact of ocular dryness and irritation symptoms on daily life. Objective evaluation of ocular surface condition will be measured for left and right eyes using Keratograph 5 M 77000 (Oculus Corporation, Germany), as described elsewhere (57). The first tear break-up time, average tear break-up time, tear meniscus height, meibomian gland function, and corneal and conjunctival fluorescein staining will be collected by the trained ophthalmologist.

##### 2.9.2.7 Retinal thickness

Retinal morphology will be assessed with OCT TR-KT-2736 (optical coherence tomography; Heidelberg Engineering Corporation, Germany), a noninvasive imaging technique that enables optical biopsy *in situ* and in real time, as described elsewhere (58, 59). Participants are seated with their head fixed at the designated position of the instrument, and are instructed to stare at the gaze target for about 10 s. Macular retinal thickness (mRT) is acquired within a volume of the macular cube 512 × 128, and ETDRS grid is used to extract the mRT corresponding to the 9 macular subfields: fovea centralis, four quadrants (superior, nasal, inferior, temporal) with radii of 1–3 mm and 3–6 mm around fovea (the inner ring and outer ring). Mean macular retinal nerve fiber layer thickness, the thickness of the retinal ganglion cell layer, and inner plexiform layer in four quadrants (superior, nasal, inferior, temporal), and retinal volume are extracted from color maps generated by the OCT software. Thirty minutes after pupil dilation with 0.5% tropicamide and 2.5% phenylephrine, OCT scans will be performed separately for each eye by an experienced ophthalmologist.

##### 2.9.2.8 Optical quality

Information regarding accommodative ability will be obtained with the double-pass retinal images of the OQAS II (the Optical Quality Analysis System; Visiometrics Corporation, Spain), including strehl ratio, modulation transfer function (MTF) cutoff frequency, object scatter index and the OQAS values. Refractive errors, corneal curvature, pupil diameter, and MTF are determined using an OPD-Scan III unit (Nidek Corporation, Japan). Images will be automatically captured and recorded on the system through a strict quality control of assay performance. All examinations are performed for each eye by an impartial ophthalmologist using standard operating procedures.

#### 2.9.3 Blood samples and serum carotenoid levels

Fasting venous blood samples will be obtained by trained nurses or phlebotomists. Two vials of whole blood will be collected from each participant: (1) 5 mL into EDTA-containing tubes; (2) 5 mL into vacutainer tubes containing clot activator. Blood samples from vial 1 are centrifuged at 3500 *g* for 15 min at 4°C within 30 min after being drawn for separating plasma, buffy coat, and red blood cells (RBCs). Blood samples from vial 2 are allowed to sit at room temperature for 60 min to coagulate, and then centrifuged at 3500 *g* for 15 min at 4°C for separating serum. The resultant samples are aliquoted in 1.5 mL well-labeled tubes, and stored at −80°C in the biobank of the Xi’an Jiaotong University until assay.

Measurements of serum concentrations of lutein, zeaxanthin, *β*-cryptoxanthin, *α*-carotene, *β*-carotene, and lycopene will be performed at the key laboratory using a reversed-phase high performance liquid chromatography (HPLC) system equipped with a YMC-carotenoid C30 analytic column (250 mm × 4.6 mm × 5 μm; YMC Corporation, Japan), as previously described (60). Cryopreserved serum aliquots are thawed in a subdued light container at room temperature. Processing of the sample involves precipitation by 250 μL of mixed internal standard solution (methanol: acetonitrile = 1:2, v/v) containing 5 μL of echinenone, dryness of the aggregate supernatant under nitrogen gas, and reconstitution by 100 μL of methanol after drying the extracts. The mobile phase is methanol (A), and isopropyl: n-hexane (1:1, v/v) (B) with a gradient elution program of 0–50% (B) in 0–20 min, 50–100% (B) in 20–30 min and then re-equilibration of the column with 0% B for an additional 8 min. The injection volume is 10 μL at a flow rate of 1 mL/min, and the column temperature is kept at 15°C. Chromatograms are evaluated at 450 nm for carotenoids by using purified external standards. Concentrations are derived using eight-point standard addition calibration curves in the range of 0.005–50 μg/mL. Quality control samples at three concentrations (low, medium, and high) are prepared for method validation.

#### 2.9.4 Erythrocyte membrane omega-3 fatty acids

The omega-3 fatty acids content of RBC membranes will be measured at the central medical laboratory by using gas chromatography (GC) technique equipped with an SP-2560 fused-silica capillary column (100 m × 0.25 mm × 0.2 μm; Shimadzu Corporation, Japan) following published methods (61–63). A typical procedure will be used for the preparation of red blood cell (RBC) membrane samples as follows: the 200 μL membrane suspension is mixed with hexane and isopropanol (2.5 mL; 3:2, v/v) containing 15 μL of eicosanoic acid methyl ester as an internal standard, and then blended sufficiently. After centrifugation, the supernatant is collected and taken to dryness under a vacuum concentration system. The dried residue is methylated with methanol and acetyl chloride (25 mL; 4:1, v/v) and then heated at 95°C for 1 h. After cooling, the sample is mixed with 2.25 mL of 48% K_2_CO_3_, and the combined supernatant is dried down under nitrogen. The resultant fatty acid methyl esters (FAMEs) are solubilized in 80 μL isooctane and 20 μL is injected into the GC system. The oven temperature is 170°C for 5 min, increased to 175°C at a rate of 5°C/min, then increased 1°C/min up to 210°C, held for 5 min, followed by a final increase of 5°C/min up to 240°C, held for 20 min. DHA and EPA are identified by comparing their peak retention times with a standard mixture of FAMEs and expressed as relative percentages of total fatty acids. A series of quality control assays are performed to determine the data repeatability.

#### 2.9.5 DNA extraction and genome-wide genotyping

High quality DNA samples will be purified from the buffy coat using the Genomic DNA Extraction kits according to the manufacturer’s instructions. Genome-wide genotyping will be carried out using the Affymetrix Axiom^®^ Array Automated Workflow, following the manufacturer’s protocol (64, 65). DNA stock solutions are amplified and fragmented into base pair fragments. These fragments are purified, resuspended, and then hybridized to Affymetrix Axiom^®^ Arrays. Hybridization, ligation, washing, staining, and scanning are automatically processed on the GeneTitan Instrument (Affymetrix). Finally, the genotypes of total genomic DNA for each sample are obtained through the Genotyping Console program (Affymetrix). Data management is performed on a High Performance Computing cluster. Quality control filters are applied to study samples and genotyping (e.g., genetic sex matching self-reported sex, per-sample missing rate < 10%), single nucleotide polymorphisms (SNPs) with minor-allele frequency > 1%, SNPs with a genotyping success rate of ≥95%, and Hardy–Weinberg Equilibrium *p*-value >1 × 10^−6^.

#### 2.9.6 Fecal sample collection and 16S rRNA gene sequencing

Stool samples are obtained by participants themselves using stool collection kits following a detailed description of sample collection instructions. All samples are immediately transferred to −80°C freezer using an insulated foam box filled with ice within 2 h of collection until subsequent analysis of intestinal microbial composition.

Total microbial DNA will be extracted from each fecal sample using the Stool DNA Purification Kit in accordance with the manufacturer’s instructions. The V3 to V4 region of the bacterial 16S ribosomal RNA (rRNA) gene will be amplified via the polymerase chain reaction technique using primers with unique barcodes (338F and 806 R), High-throughput sequencing of the amplified products is performed utilizing an Illumina MiSeq sequencer (66). The quality filtering of raw sequences and operational taxonomic units (OTUs) clustering are conducted by the Quantitative Insights into Microbial Ecology (QIIME) platform following the standard operating procedure. All effective paired-end reads from each sample are assigned to the same OTUs based on a 97% identity threshold. Metrics of alpha diversity, including the standard Shannon and Simpson diversity indices and richness indices (the abundance coverage-based and the Chao1 estimator) will be calculated based on the rarefied OTU counts. Principal component analysis and principal coordinate analysis are performed to evaluate the beta diversity of bacterial flora in samples.

#### 2.9.7 Covariates

Information on contact details (name, phone number, and place of residence), demographics (age, sex, educational attainment, employment, parental education, and household income), daily screen time of near-field technological devices (smartphones and computers), reading habits (the use of a table lamp, reading distance, and performing eye exercises), sunlight exposure (residence calendar, employment history, time spent outdoors during work and leisure activities, and hat and eyeglass use), lifestyle behaviors (smoking, alcohol consumption, the use of antioxidant supplement, physical activity, and sleep quality), family history (cardiovascular disease, diabetes mellitus, myopia, glaucoma, cataract, and AMD), and menstruation will be collected using standardized questionnaires by trained fieldworkers. Screen time utilization and the use of electronic devices in a dark environment at night, will be evaluated by the time spent on smartphones and computers on typical weekdays and weekends. The mean daily screen time will be calculated using the formula: (5 × hours of screen time on weekdays +2 × hours of screen time on weekends)/7. The validity of the questionnaires in measuring smartphone use was evaluated by comparing with data from the preinstalled application located in the smartphone’s settings in a subsample of the participants in this trial (*n* = 30). Correlation coefficient between subjective and objective measures was 0.55 for daily smartphone use. The ocular ambient exposure to different wavelengths of light will be constructed on the basis of detailed exposure histories and published ambient data. Participants will be asked to provide information on residential history, work history, the average time spent outdoors during daylight hours for different occupational and leisure periods in winter and summer, and the proportion of hat and eyewear (glasses, contact lenses, and sunglasses) use after the age of 15 years (67, 68). Ambient data will be obtained from the National Aeronautics and Space Administration Goddard Earth Sciences Data and Information Services Center (69), which provides data on erythemally weighted daily dose of ultraviolet (UV, J/m^2^), UV index, and noontime spectral irradiances at four wavelengths (305, 310, 324, and 380 nm). Geographical coordinates of each location are used to assign the daily ambient exposure levels to each subject. Blue light (400 to 500 nm) in the visible spectrum will be estimated using the tropospheric ultraviolet and visible radiation transport model (70). Yearly ocular exposure to each wavelength of light will be quantified using an adaptation of the mathematical model developed by Rosenthal et al. (71), by summing the daily fraction of ocular exposure during work and leisure hours. The daily fractions of ambient radiation reaching the eye are determined from ambient exposure at each place, the time spent at that location, the ocular ambient exposure ratio, and attenuation factors provided by ocular protection (72, 73). Finally, a cumulative ocular exposure is calculated as the sum of the yearly exposures. Physical activity will be assessed using a validated International Physical Activity Questionnaire (74). Subjective sleep quality will be evaluated by a validated Pittsburgh Sleep Quality Index (75).

#### 2.9.8 Anthropometry

Anthropometry will be measured in triplicate by trained staff and the average reading is recorded. Systolic and diastolic blood pressure are measured in a seated position after 10 min of resting using an automated digital device (Omron Corporation, China). Body weight and composition are measured without shoes by bioelectrical impedance using a calibrated BC-582 digital scale (Tanita Corporation, China). Body mass index (BMI; in kg/m^2^) is calculated as kilograms (kg) of weight divided by the squared height in meters (m) and categorized as “underweight (<18.5 kg/m^2^),” “normal weight (18.5–24.9 kg/m^2^),” “overweight (25–29.9 kg/m^2^),” and “obese (≥30.0 kg/m^2^),” defined by World Health Organization described by Global BMI Mortality Collaboration (76). Waist circumference will be assessed at the umbilicus to the nearest 0.1 cm using a non-elastic measuring tape.

#### 2.9.9 Dietary assessment

Dietary intake is assessed using a semi-quantitative food frequency questionnaire (FFQ) by trained research staff to collect information on the consumption frequency in standard portion sizes of each food item over the previous year. The FFQ consists of 105 food items which are assembled into 12 categories: cereals; vegetables; legumes and soybean products; mushrooms and seaweeds; fruits; nuts; egg, meat and processed meat; milk and dairy products; beverages; snack foods and confectionaries; oils; and condiments. Participants will be requested to choose from nine predefined categories of preidentified foods as follows: “never or less than once per month”; “1–3 times per month”; “once a week”; “2–4 times per week”; “5–6 times per week”; “once per day”; “2–3 times per week”; “4–6 times per week”; and “6 or more times per day.” Consumption frequencies are converted into the daily intake frequencies of the given food item using the midpoint of frequency options. Daily intakes of energy and nutrients are computed by multiplying the daily intake of each food item (g/d) with the relevant nutrient in specified portions (100 g), summed across all foods consumed. The reproducibility and validity of this FFQ have been documented in previous studies performed by our research group (77).

### 2.10 Safety assessments

A safety assessment including adverse events (AEs) and concomitant medications will be conducted from baseline until the final visit or early withdrawal. AEs are defined as any untoward medical occurrence among participants, which are not necessarily associated with supplement use. Participants will be advised to report any unfavorable and unintended symptom, sign or disease to the research assistant as soon as they can by e-mail or telephone during the intervention. In addition, abnormal findings considered clinically significant by a medical staff will be also recorded as an AE. Serious adverse events are defined as any untoward medical occurrence that caused hospitalization, significant incapacity or permanent damage, and any other serious medical condition requiring medical or surgical intervention. Information regarding the course of AEs, including description, date of onset, duration, actions taken, and outcome, is registered using a standardized form and evaluated for severity and causal relation to the study treatment by a medical investigator.

### 2.11 Data management and confidentiality

Data management and confidentiality will be performed by the Xi’an Jiaotong University Health Science Center. At enrollment, proper measures will be taken by keeping the identity of the participant anonymous by replacing their full names and contact details with a unique number. Data collection forms in paper-based format will be securely stored in a locked cabinet with restricted access only to designated study members. Survey questionnaires will be completed and stored electronically in the Research Electronic Data Capture (REDCap) system, an online secure data management system hosted by the Xi’an Jiaotong University. REDCap servers are encrypted and accessible only by authorized personnel. In some cases, representatives from BYHEALTH Co., LTD and Xi’an Jiaotong University Health Science Center will review the accumulating data for the purposes of monitoring safety and quality of data and the fidelity of statistical analysis.

### 2.12 Statistical analysis

Analysis of the primary and secondary outcomes will be conducted following the intention-to-treat (ITT) principle, whereby participants will be included in the groups to which they were originally assigned, irrespective of compliance or loss to follow-up. Missing values on baseline covariates and follow-up data will be imputed by multiple imputation methodology (78). Investigators and participants remain blinded to treatment assignment during the trial. No interim analysis is planned.

Normally distributed values will be assessed and verified using the Shapiro–Wilk test. Descriptive summaries of quantitative variables include means and standard deviations for normally distributed variables and interquartile ranges for nonnormal variables. Categorical variables are presented as frequencies and percentages. Baseline characteristics of each arm will be compared by the two-sample t-test, one-way analysis of variance (ANCOVA) and the Wilcoxon rank-sum test for continuous variables, and chi-squared test for categorical variables.

Change in continuous outcomes at each time point will be calculated as follow-up minus baseline and be represented as the mean change and 95% confidence intervals. Change in primary and secondary outcomes from baseline at 24 weeks between study arms will be performed using ANCOVA adjusted for potential covariates. Paired t test and Wilcoxon signed-rank test will be conducted for normally and non-normally distributed data, respectively. Linear mixed effects models will be performed to evaluate differences in outcome changes over time (e.g., baseline, 12, and 24 weeks) between the intervention and control groups, with the baseline values of corresponding outcomes, stratifying variables and time-group-interactions as covariates. Multiplicative interaction terms in linear mixed effects models are used to explore interactions between genetic variations or gut microbiota and the intervention outcomes.

Sensitivity analyses will be performed to verify the robustness of the results by converting continuous outcomes into categorized values and using generalized estimating equations comparing the risk ratio between groups. Subgroup analyses will be conducted to assess the moderation of the effects of randomized intervention groups by the baseline covariates (e.g., sex, time spent outdoors, ocular exposure to ambient UV radiation, blue light exposure, screen time of digital devices, or dietary carotenoid intake).

Statistical analyses are performed using SPSS statistical package version 22.0, and R version 3.4.0 (e.g., the lme4 package, and the lmerTest package), and a two-sided *p* ≤ 0.05 will be used to determine statistical significance.

## 3 Discussion

The LZO study is to determine whether 24 weeks of supplementation with lutein, zeaxanthin, and omega-3 fatty acids can improve MP and visual function in young adults with long-term screen time exposure. With the ubiquitousness of screen-based technology and the accessibility of the internet, a dramatically rapid increase in screen viewing has been observed in young adults, which comes up with a radically increased in the prevalence of various blindness-causing eye diseases (5, 7). The retina is under considerable oxidative stress and extremely vulnerable to photochemical damage due to repeated exposure to light and the highest metabolic rate in the retina (30, 35, 79). Prolonged near-field exposure to the relatively high-energy, short-wavelength light tends to cause a series of long-term, cumulative alterations, including irreversible cellular damage of photoreceptors and RPE arising from increased oxidative stress, low-grade chronic inflammation (e.g., interleukin 1, tumor necrosis factor-*α*, monocyte adhesion factor), and the suppression of mitochondria and lysosome function (8, 80–82). Thus, this study may have far-reaching implications for developing early interventions to delay the onset of blue light-induced photo-oxidative damage in young adults.

As the main pigments deposited in the macula, lutein and zeaxanthin have been demonstrated to function as blue light filters and antioxidants (13), and have favorable effects on biological processes involved in AMD pathogenesis (83). Previous clinical studies have demonstrated the benefits of daily supplementation with macular xanthophylls for improving MPOD and visual performance (19–21). A recent meta-analysis of 43 RCTs has addressed the response to carotenoid-rich food or supplements of MP and VA in both healthy population and patients with eye disease, such as AMD, cataracts, and glaucoma (19). However, little is known regarding their efficacy in young adults with long-term repeated exposure to digital displays (7, 22, 23). Our previous study reported a protective effect of supplemental intake of 12 mg/d lutein for improvement of CS and GS in 37 healthy subjects with longer use of digital devices (22). Given the potential deleterious effects of the dramatic increase in the use of near-field electronic devices, further exploration of nutritional intervention with larger sample size and various supplementation regimens is urgently needed in young adults with high screen exposure. Our current clinical trial is designed to generate a rich data set of comprehensive eye-health parameters, so as to provide evidence about the effects of administration of lutein and zeaxanthin on various visual outcomes. Moreover, zeaxanthin preferentially accumulates in the fovea of the macula with especially high concentrations, whereas lutein is diffusely localized across the retina at relatively lower concentration (24). The specific distribution pattern implies that zeaxanthin may exert unique visual benefits on macular morphology. Nevertheless, the results of clinical studies have been inconsistent (84, 85).

A thorough understanding of lutein bioavailability in relation to chemical structural complexity is necessary. Lutein can be found in the free form or esterified with fatty acids in the diet. The ester forms require prior de-esterification by intestinal enzymes and are converted into free lutein before being taken up by the enterocyte (86). Data regarding supplemental intake of free lutein or lutein esters on serum response and MPOD have shown diverging results (25–29). A study by Yoshizako et al. (28) investigated the effect of free lutein and lutein esters supplements in healthy Japanese subjects and reported significant elevations on MP and serum lutein concentration in response to 10 mg of free lutein and 20 mg of lutein esters (equivalent to 10 mg of free lutein), however no significant differences were found between the two groups. In contrast, Norkus et al. carried out a clinical trial in 72 volunteers, and noted the relative serum lutein response was significantly greater from supplements containing 12.2 mg of free lutein than from supplements containing 27 mg of lutein esters (equivalent to 13.5 mg free lutein) (26). In an RCT with bioavailability as the primary outcome, 18 subjects randomly assigned to the lutein diester formulation (equivalent to 20.7 mg free lutein) or the unesterified formulation (equivalent to 24.5 mg free lutein) showed that lutein esters were 61.6% more bioavailable than the unesterified lutein formulation with higher maximum serum concentration and ascending slope (25). The LZO trial will be performed to provide more knowledge on the bioavailability of free lutein and lutein ester.

The evidence for the synergistic effects of supplemental intake of xanthophyll carotenoids and omega-3 fatty acids on the visual system is similarly unclear. DHA is a key fatty acid accounting for over 50% structural lipid in the phospholipid fatty acyl chains of retinal POS (31, 33). Putative mechanisms for beneficial effects for DHA include maintaining proper disc morphology, stimulating POS membrane biogenesis and subsequently phagocytosis by the RPE, and interacting with rhodopsin (87, 88). Furthermore, DHA can be enzymatically converted into protective mediators (e.g., docosanoids, elovanoids), serving as neuroprotective signaling for homeostatic regulation of cell integrity in the retina (89). As a substrate for DHA, EPA has the potential to influence AA-based eicosanoids metabolism, implicated in abnormal neovascularization and inflammation in the retina (90). Based on their biological function, it is likely that DHA and EPA play crucial roles in the pathogenesis of AMD. Recently, clinical trials have shown the association between supplemental omega-3 fatty acids and improved changes in lipoprotein profile, which may promote xanthophyll transport to the retina (91, 92). However, data is insufficient to draw any definite conclusions. The results of our study will make a substantial contribution to the evidence gap about possible additive effects of carotenoids and omega-3 fatty acids supplementation on MP and visual function.

Evidence regarding the wide-ranging interindividual variability in response to nutrient supplementation has been well described in previous studies (93–95). Similarly, the intestinal uptake or efflux, absorption, metabolism, and tissue response of dietary carotenoids and omega-3 fatty acids are particularly influenced by ethnic origin, genetic variations, and gut microbiota (38, 39). Genetic variants in genes encoding for numerous proteins involved in carotenoid metabolism could modulate the bioavailability of dietary carotenoids by affecting the expression or activity of these proteins (96, 97), which would explain the interindividual variability of the differential visual improvement achieved by individuals assigned to the same intervention. Moreover, a recent systematic review of animal studies suggests the association between carotenoids and intestinal bacteria related to both inflammation and short-chain fatty acid production (98), prompting that the interrelationship between gut health and dietary carotenoids may play a pivotal role in the initial of retinopathies. Therefore, an additional goal of this trial is to assess the impact of supplementation with xanthophyll carotenoids and omega-3 fatty acids on host intestinal health, and explore whether genetic variations, gut microbiota composition, and changes in intestinal microbiota interact with the dietary effects on MPOD and visual performance, seeking underlying mechanistic insights into interindividual differences.

It is well-established that excessive exposure to light radiation emitted from the sun could induce deteriorative changes to the retina, which have been associated with retinal degenerative diseases. In a Müller glia lineage-tracing mouse model of photo-induced retinal injury, the light-exposed groups significantly increased profound ROS production in the retina, and exhibited a dramatically declining trend in a-wave and b-wave amplitudes (99). *In vitro* studies have revealed that prolonged UV irradiation led to the imbalance of oxidative stress, lipid peroxidation, along with mitochondrial depolarization and DNA damages, which eventually caused apoptosis of RPEs and retinal ganglion cells (100, 101). Although findings from epidemiological evidence of the association between sunlight exposure and AMD are not consistent (102–107), the European Eye Study found that higher exposure to blue light from sunlight was associated with an increased risk of AMD in participants in the lowest quartile of blood antioxidant levels of vitamin C, vitamin E, and zeaxanthin (105), suggesting the importance of supplementation with carotenoids in the population with long-term exposure to blue light and suboptimal levels of lutein and zeaxanthin.

Several limitations should be considered. Firstly, considering the eligible population is restricted to those who are aged 18 years or older and use digital devices for more than 8 h daily, the generalization of the findings to other populations is limited. Secondly, screen time utilization is gathered using the self-reported questionnaire, which may skew toward conservative properties and underestimation (108). Potential recall bias cannot be completely eliminated. Nevertheless, subjective estimations of screen exposure were positively correlated with data from smartphone applications in previous studies (109), supporting the reasonable validity for the assessment of digital screen exposure. Objective measurements of near-vision work activities are needed specifically to precisely track real-time use of digital devices in future studies.

In summary, we hope that the implementation of the LZO trial may assist in developing early intervention strategies to protect the morphological integrity of the retina and prevent the onset of irreversible blindness, and contribute to the development of precision nutrition interventions for sensitive populations with long-term cumulative exposure of blue light emitted from electronic devices.

## 4 Ethics and dissemination

### 4.1 Ethics approval and consent to participate

The study was approved by the institutional review board at the Xi’an Jiaotong University (2019–1288). The trial was declared and registered at the Thai Clinical Trials Registry (TCTR20220904002). Recruitment of participants started in July 2019 and lasted for 1 year. A detailed verbal description of the study purposes, procedures, the benefits, and potential risks will be provided to each individual, and written informed consent will be collected from all participants following the guidance from investigators prior to participation. The information form will contain purpose and plan of the research, possible risks and benefits, ethics committee approval, statements on voluntary participation, the right to withdraw from the study at any time, confidentiality and privacy arrangements, and consent provisions for the collection and use of data and biological specimens. Participants will not be permitted to attend any screening process until the written informed consent has been signed.

### 4.2 Protocol amendments

Any protocol modifications will be formally submitted to the institutional review board of the Xi’an Jiaotong University and trial sponsors. Protocol amendments will be updated in the Thai Clinical Trials Registry accordingly and reported to all study investigators.

### 4.3 Dissemination policy

The research findings will be disseminated through peer-reviewed journals and conference presentations.
